# Engineered reversal of drug resistance in cancer cells—metastases suppressor factors as change agents

**DOI:** 10.1093/nar/gkt946

**Published:** 2013-10-23

**Authors:** Vinod Kumar Yadav, Akinchan Kumar, Anita Mann, Suruchi Aggarwal, Maneesh Kumar, Sumitabho Deb Roy, Subrata Kumar Pore, Rajkumar Banerjee, Jerald Mahesh Kumar, Ram Krishna Thakur, Shantanu Chowdhury

**Affiliations:** ^1^G.N.R. Knowledge Centre for Genome Informatics, ^2^Proteomics and Structural Biology Unit, Institute of Genomics and Integrative Biology, CSIR, Mall Road, Delhi 110 007, India, ^3^Biomaterials Group, Indian Institute of Chemical Technology, Hyderabad 500 607, India and ^4^Animal House, Centre For Cellular and Molecular Biology, Uppal Road, Hyderabad 500 007, India

## Abstract

Building molecular correlates of drug resistance in cancer and exploiting them for therapeutic intervention remains a pressing clinical need. To identify factors that impact drug resistance herein we built a model that couples inherent cell-based response toward drugs with transcriptomes of resistant/sensitive cells. To test this model, we focused on a group of genes called metastasis suppressor genes (MSGs) that influence aggressiveness and metastatic potential of cancers. Interestingly, modeling of 84 000 drug response transcriptome combinations predicted multiple MSGs to be associated with resistance of different cell types and drugs. As a case study, on inducing MSG levels in a drug resistant breast cancer line resistance to anticancer drugs caerulomycin, camptothecin and topotecan decreased by more than 50–60%, in both culture conditions and also in tumors generated in mice, in contrast to control un-induced cells. To our knowledge, this is the first demonstration of engineered reversal of drug resistance in cancer cells based on a model that exploits inherent cellular response profiles.

## INTRODUCTION

Acquisition of resistance toward drugs is detrimental to targeted cancer therapy. It is one of the major roadblocks in treatment toward several malignancies. Various mechanisms implicated in drug resistance of tumor cells include activation of drug efflux pumps, increased drug metabolism, epithelial to mesenchymal transition (EMT) and secondary mutations in drug target(s) (1). Recent work catalogs vast number of cell–drug response associations (2), and molecular correlates derived from gene expression signatures predict new targets (3), mode of action pathways (4) and suggest drug repositioning, demonstrating the effectiveness of integrative approaches (4–8). For example, Kutalik *et al.* (6) showed co-modules in large and ‘noisy’ gene expression data sets can be used to integrate multiple parameters and produce more coherent patterns; in another approach drug and gene expression data from the Connectivity Map were used to make networks of response information which segregated in a mode of action-dependent fashion (4). Recently, this approach was further and substantially extended by three studies. First, Barretina *et al.* (5) reported response data of 479 cancer cell lines for 24 anticancer drugs along with sequence of the cancer lines. Second, in another study response of 639 tumor cell lines toward 130 clinical/preclinical anticancer molecules was studied along with the mutational spectrum of 64 commonly mutated genes in cancer (9) and third, Wacker *et al.* (10) used transcriptome sequencing to identify mechanisms of drug action and resistance and as proof-of-concept cytotoxic anticancer drugs BI 2536 and bortezomib were studied in detail.

Emerging observations draw interesting correlations between EMT, a differentiation program essential for morphogenesis during embryo development, and incidence of drug resistance (reviewed in Singh *et al.* (1)). The process of EMT is regulated by growth factors and cytokines including transforming growth factor (TGF)-beta, however this evolutionarily conserved developmental program can be deregulated in cancer cells during tumor progression resulting in gain of not only invasiveness and metastatic characteristics but also resistance to drugs (11,12). Intriguingly, recent evidence suggests that EMT can induce reversion to a cancer stem cell (CSC)-like state (11,12), further associating EMT, CSC and drug resistance. Incidence of metastasis involves several stages/processes, including gain of invasive characteristics (mesenchymal cells resulting from EMT), intravasation and survival in circulation, homing and extravasation at the secondary organ site followed by reacquisition of ‘epithelialness’ (which involves mesenchymal to epithelial transition) and subsequent colonization (13–15). Given this complexity it is intriguing that a class of factors has been discovered that can negatively impact the metastatic process, and are called metastasis suppressor genes (MSGs). By definition, MSG function is described in the context of dissemination of cells from the primary site of tumor incidence to distant sites (reviewed in (16,17)), a process that is understood to contribute to more than 90% of cancer-associated mortality (13,15), thereby underscoring the importance of MSG function in tumor cells.

Together these studies provide a vast framework for associating molecular processes that can drive occurrence of drug resistance in cancer cells, however, it is still not clear whether this can be extended beyond associations to engineer resistance in cancer cells. With this in mind, we sought to study transcriptomes of cancer cells along with drug response data to identify appropriate molecular factors. Our approach invokes and experimentally tests models based on gene signature modules for re-engineering inherent response of cancer cells toward drugs. Proof-of-concept findings presented here build support for engineered reversal of resistance vindicating the use of high throughput transcriptome and drug response profiles, and therefore, extend the scope of such data for re-engineering approaches.

## MATERIALS AND METHODS

### Chemosensitivity and MSG expression analysis

The 60 cell lines used in this study were previously assayed for their sensitivity to a variety of compounds as a part of the Developmental Therapeutics Program at the National Cancer Institute, as described (http://dtp.nci.nih.gov) (18). For this work, first GI50 of all 60 cell lines were clustered for the 1400 drugs and then the expression values of all cell lines were clustered for 30 MSGs. Next, the cell lines were classified as resistant or sensitive for each compound. Cell lines with GI50 (log10) at least 0.8 SDs above the mean were defined as ‘resistant’ to the compound tested, whereas those with GI50 (log10) at least 0.8 SDs below the mean were defined as sensitive. Analysis was performed for compounds with at least six sensitive and six resistant cell lines. For all drug molecules that had at least six cell lines in each group the expression of MSGs were noted and averaged to produce a representation that shows the effect of all genes vis-a-via drug molecules. The cell groups were further used to produce drug signatures to be compared with the experimental gene expression profiles. Two-dimensional unsupervised hierarchical clustering was performed using cluster software developed by Eisen labs (19), and heat map was generated using Treeview software (20).

### Analysis of gene expression signatures

To test correlation between gene expressions differences observed in drug response versus the changes observed on inducing the MSG Non-Metastatic 2 (NME2), we first pre-processed the data sets using a previously published method (21). Briefly, *v* (defined as relative fold change for each gene) between expression in resistant versus sensitive cell groups for any given drug molecule was calculated using

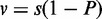

where ‘*P*’ is the *P*-value calculated by performing a student’s *t*-test for expression difference of a gene between the resistant and sensitive cell lines, ‘*s*’ denotes the sign of the difference between the average values in the two sets considered. Therefore, ‘*v*’ indicates the extent to which a gene was up- or down-regulated in the resistant cell lines relative to sensitive ones with maximal and minimal values of 1 and –1, respectively. ‘*v*’ for genes from the data set of NME2-induced MDA-MB-231 cells was calculated similarly using respective un-induced cells as control. Following this, ‘*v*’ obtained from drug response was compared with that from NME2-induced condition for respective genes; statistical significance of this comparison was tested using the Pearson’s correlation test. Differentially expressed genes were analysed using Genecodis2 (www.genecodis.dacya.ucm.es) to identify enriched pathways and biological processes.

### Cells and culture conditions

MDA-MB-231 and MDA-MB-468 cells were obtained from the national repository of cell lines at National Centre for Cell Sciences (NCCSs), Pune, India and maintained in Dulbecco’s modified Eagle medium (DMEM) supplemented with 10% fetal bovine serum at 37°C in 5% CO_2_ environment. The cells were transfected using NME2-GFP/only GFP cDNA (Origene Inc., USA) plasmid vector containing a CMV promoter for constitutive expression of NME2-GFP fusion protein, and stable clones were selected using G418 sodium salt.

### Gene expression profiling

Total RNA was purified from MDA-MB-231 cells expressing GFP alone (vector control) or NME2-GFP fusion protein using TRIzol reagent (Sigma, USA). mRNA was linearly amplified by *in vitro* transcription using T7 RNA polymerase (MEGA script T7 kit, Ambion, Inc., USA). The quality and integrity of total and amplified mRNA (cRNA) was monitored by both spectrophotometry (OD UV 260/280 ratio > 1.8) and Agilent bioanalyser. Gene-expression profiling was performed using Illumina HumanWG-6 BeadChip, which contains 47 296 transcripts. BeadChips were scanned with a BeadStation 500 GX and data were analysed using bead studio software. FDR was calculated based on method proposed by Storey and Tibshirani (22).

### Drug sensitivity assay

Both MDA-MB-231 cells with GFP or NME2-GFP were plated in 96-well microtiter plates at a density of 8000 cells/well for 24 h. Both cell types were treated with anticancer drugs camptothecin, topotecan and caerulomycin at different concentrations and also with DMSO as vehicle control. After 48 h of drug treatment total number of viable cells was assayed using CellTiter-Glo® luminescent cell viability assay kit (Promega). Luminescence obtained from cells treated with DMSO was taken as 100% and percentage decrease in luminescence was calculated in response to drug doses in respective cases and data were plotted/represented as percentage cell viability.

### *In vivo* tumor growth and cell viability assay

Three million cells of each type (MDA-MB-231 breast cancer cells expressing GFP only or induced with NME2) were diluted in 150 µl of PBS and implanted in 4^th^ mammary fat pad of BALB/c nude (nu/nu) female mice. The growth of the tumor was monitored every week by a caliper. We expressed the growth as mean of tumor diameter. After 8 weeks, mice were euthanized according to institutional animal ethics protocol, and tumors were recovered and processed for histology or cell culture. For drug sensitivity assay experiments, tumors were digested in collagenase II, and the recovered cells were cultured *in vitro*. These cells were used for cell viability assays within 2–3 passages as described earlier.

## RESULTS AND DISCUSSION

**Figure 1.**
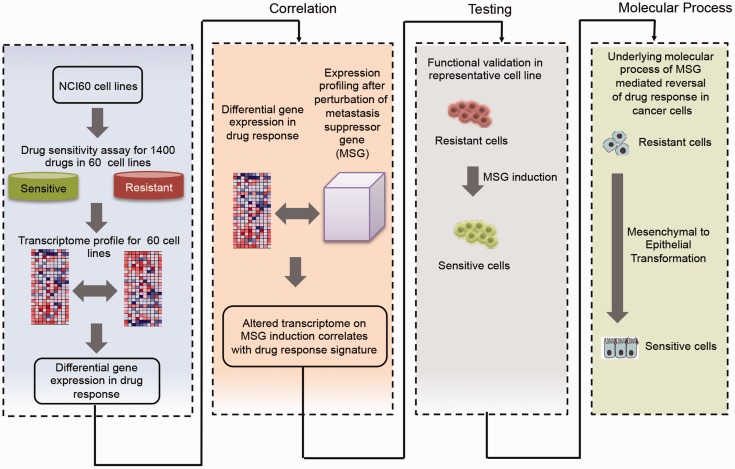
Scheme of overall approach showing coupled analysis of transcriptome profiles obtained from resistant/sensitive cells and following induction of MSG in cancer cells.

The overall approach was broadly divided into four parts (Figure 1). In the first part of this study transcriptome and drug sensitivity data from the NCI60 panel were used to build drug-specific MSG signatures. This led to the identification of differentially expressed MSGs in response to drugs. In the second part, stable induction of a candidate MSG was done in drug resistant cancer cells. Changes in transcriptome brought about by induction of MSG were compared with the drug resistance signature built from the NCI-60 panel. In the third part, we experimentally tested response of commonly used drugs in cancer therapeutics toward MSG-induced resistant cancer cells. Finally, in the fourth part we investigated the underlying molecular process of MSG-mediated reversal of drug response in cancer cells.

### Metastasis suppressor factors are repressed in drug resistant cancer cell types

**Figure 2.**
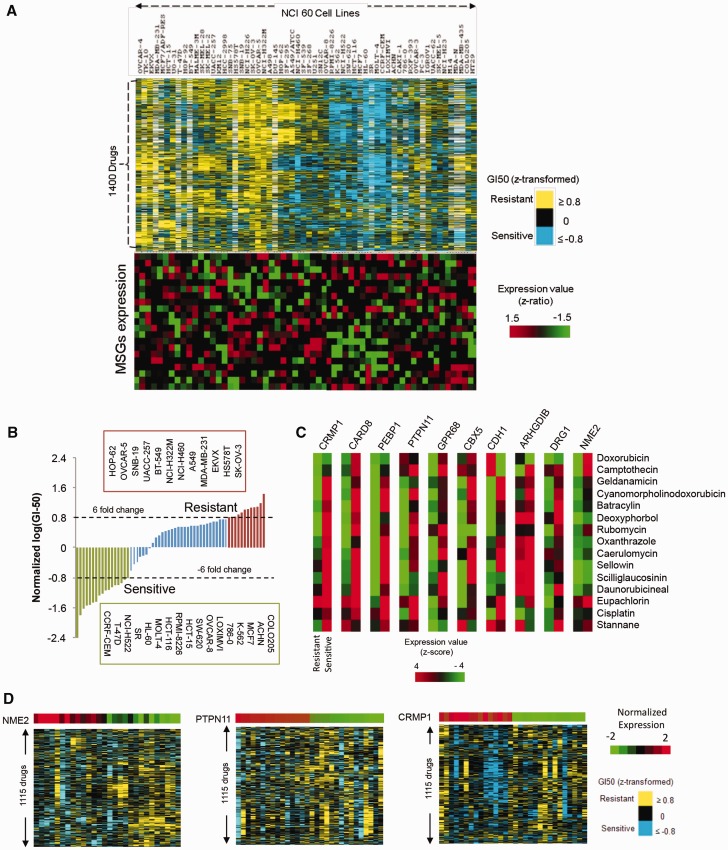
MSGs are associated with chemosensitivity. (**A**) Clustering of drug/cell line combination based on GI_50_ values (upper panel) and MSG expression in different cell lines (lower panel)—MSG expression segregates with resistance/sensitivity for many drug–cell line pairs. Absence of GI_50_ value in upper panel heatmap represented by white color. (**B**) Representation of the groups containing resistant and sensitive cell lines based on GI50 values. A cell line with at least 6-fold change above/below average GI50 was considered as resistant/sensitive, respectively. (**C**) Expression of MSG was distinct between resistant and sensitive groups of cells for many drug molecules (see Supplementary Figure S1 for all molecules). (**D**) Cell lines with high or low expression of MSG (NME2, PTPN11 or CRMP1) show distinct response to most drug molecules.

We noted many cell types despite distinct tissue origin have similar resistance/sensitivity profiles against a number of drug molecules. We first designated a cell line resistance or sensitive based on its 50% growth inhibition (GI50) for a drug, and then clustered 60 different cell types according to their reported GI50 for 1400 drugs (available from http://dtp.nci.nih.gov; Figure 2A, upper panel). To check the expression status of MSGs in sensitive/resistant cell types we selected 36 established MSGs (16,23,24). Several MSGs were repressed in resistant cell types relative to sensitive ones (Figure 2A, lower panel). We next checked whether expressions of MSGs were relatively robust across multiple cell types that were either resistant or sensitive to a particular drug. Cell types were considered sensitive or resistant if its GI50 was at least 6-folds below or above, respectively, the average GI50 for the drug across all cell lines tested (Figure 2B). Based on this, out of 1400 we found 1115 drug molecules with at least six cell lines that were resistant and also, at least 6 other cells lines that were sensitive. Next we asked whether MSG expression was significantly different between the resistant and sensitive groups for each of the 1115 drugs. Interestingly, in all the cases we found expression of at least one MSG was significantly different between the resistant and sensitive cell groups (*P* < 0.05); representative cases with higher expression of MSG in sensitive relative to resistant cell types is shown in Figure 2C (data with all MSGs and 1115 molecules is given as Supplementary Figure S1).

We further asked how differential expression of all the 36 MSGs as a group correlated in the sensitive versus resistant cell lines. This was done using 10 randomly selected sets of 36 genes; each set was checked across resistant versus sensitive cell lines for all the 1115 cell–drug combinations, and the average number of genes found differentially expressed was taken as ‘random expectation’. Significance of genes differentially expressed in the set of 36 MSGs with respect to ‘random expectation’ was found using the chi-square test. In 666 out of the 1115 (59%) cases we found a statistically significant difference in the number differentially expressed MSGs between the sensitive and resistant cell lines (*P* < 0.05).

To further test the relationship between expression of MSG and drug response we ranked 60 cells types according to expression of particular MSGs. Top 15 (high MSG expression) along with bottom 15 cell types (low MSG expression) were selected and response of these cells toward 1115 drugs was checked. Strikingly, in most cases we noted an overall reversal of the chemoresistance in cancer cells with relatively increased expression of the MSG; in order to focus on MSGs that could negatively impact resistance toward one or more drugs we show representative examples for several MSGs NME2, PTPN11 and CRMP1 (Figure 2D) CTGF, GSN and CBX5 (Supplementary Figure S2).

### Transcriptome signature derived from cancer cells induced with metastasis suppressor factors suggest altered response to drug resistance

Next we sought to test whether induction of selected MSGs can regulate drug response in cancer cells. Our approach involved: (i) derivation of expression profiles following induction of a particular MSG; (ii) construction of drug-specific expression signatures for that particular MSG derived from analysis of multiple resistant cell lines against sensitive cell lines; and finally (iii) comparison of (i) versus (ii) to test how change in MSG levels could affect drug response.

For proof-of-concept studies we selected the metastasis suppressor NME2 as the candidate MSG based on multiple considerations: NME2 levels were negatively correlated with resistance in multiple cell/drug combinations (Figure 2D) and therefore presented a potential opportunity to increase sensitivity of cells on its induction, and we found several other studies demonstrating NME2 levels to be depleted in breast, oral and ovarian carcinoma (25–28). Additionally, we analysed expression of the 36 MSGs across 2468 clinical transcriptome profiles representing four cancer types (lungs, breast, ovarian and colon) and noted that only NME2 was down-regulated consistently in advanced stages in all the cases across four cancer types (Supplementary Figure S3A and B). We selected camptothecin and doxorubicin as drugs for case studies as these have been widely studied and used against multiple cancers (29).

**Figure 3.**
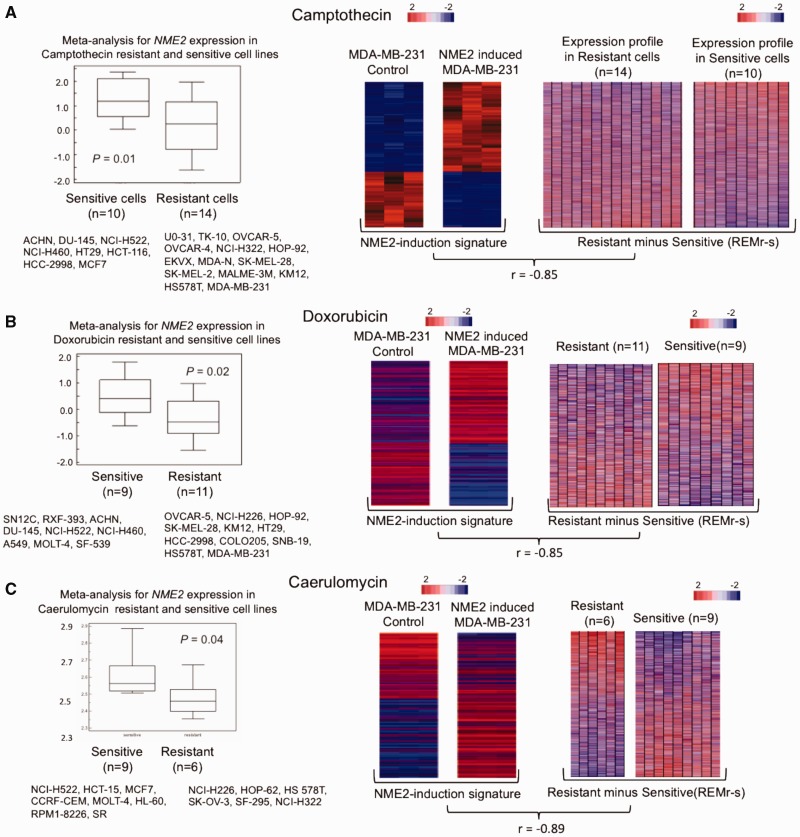
Chemoresistance and ‘MSG-induction’ are anti-correlated in many cases. (**A–C**) Comparative analysis of the ‘MSG-induction’ signature versus drug-specific gene signatures (REM)-NME2 expression in cell lines grouped as resistant or sensitive is shown in left panels. Expression profiling of MDA-MB-231 cells before or after NME2-induction and the resultant change in transcriptome of MDA-MB-231 cells is compared with the sensitive/resistant gene signature derived for camptothecin (A), doxorubicin (B) and caerulomycin (C) in the right panels. Correlation of the resistant-minus-sensitive gene signature derived from multiple cell lines—response engineering module (REM*_r_*_–_*_s_*)—with the ‘NME2-induction’ signature in MDA-MB-231cells (center panels) is shown.

We checked NME2 levels in cell types grouped as resistant or sensitive with respect to the drug camptothecin. We observed lower level of NME2 in resistant cell types compared with sensitive cell types suggesting levels of MSG could be crucial in determining response of drugs in cancer cells (Figure 3A). From the list of resistant cell types we selected the widely used metastatic breast cancer line MDA-MB-231 which results in aggressive drug resistant tumors (30). To test the effect of NME2 on the drug response, we performed gene expression profiling of MDA-MB-231 cells before and after NME2-induction; 1522 genes were up and 331 genes down-regulated (*P* < 0.01; FDR < 10%)—this differential expression profile was designated as the ‘NME2-induction’ signature (Figure 3A). Based on our prediction we conjectured that NME2-induction would result in enhanced response toward a drug, and therefore expected the ‘NME2-induction’ signature obtained in MDA-MB-231 to correlate negatively with the gene expression signature found in cells resistant to camptothecin. To test this we build an expression signature that would define resistant versus sensitive ‘states’ from a group of cell types.

### Drug-specific signatures of resistance constructed from multiple cell types and tested for re-engineering drug response

Drug-specific gene expression signatures were built based on: (i) average expression of constituent genes of the signature was significantly different in sensitive cell types relative to resistant ones (at least 6 cell types were considered in each category sensitive/resistant); and (b) in addition, expression of each gene was required to significantly correlate with GI50 values. In other words, for a given drug and say, the resistant cell types, expression of the gene would increase or decrease in a consistent fashion with respect to the increase/decrease (or vice versa) in GI50 (Supplementary Figure S4). Using 10 different cell types that are sensitive and 14 cell types that are resistant to camptothecin we therefore built a camptothecin-specific resistance-minus-sensitive expression signature and called this the response engineering module, resistance-minus-sensitive (REM*_r_*_–_*_s_*) (Figure 3A). This was done so that we could define a minimal signature for each drug on a case-to-case basis and then identify factors that significantly perturb the REM. The ‘NME2-induction’ signature was found to negatively correlate with the camptothecin REM*_r_*_–_*_s_* (*r* = –0.85, *P* < 0.001, see Methods section for details of the analysis).

In a similar fashion, we next analysed the ‘NME2-induction’ signature against the doxorubicin REM and found this to negatively correlate with the resistance-minus-sensitive signature (*r* = –0.85, *P* < 0.001, Figure 3B).

Resistant and sensitive group of cells were constructed based on arbitrary GI50 cut-off of 6-fold change. To test whether the effect extends beyond this threshold we studied response of NME2-induced MDA-MB-231 cells toward the anticancer molecule caerulomycin (MDA-MB-231 cells were not found in the list of resistance cells according to our GI50 criterion, Figure 3C). We observed negative correlation between the caerulomycin REM*_r_*_–_*_s_* and ‘NME2-induction’ signature (*r* = –0.89, *P* < 0.001, Figure 3C), suggesting that this model works beyond the applied threshold effectively. Additionally, we compared REM*_r_*_–_*_s_* for three other drugs with the ‘NME2-induction’ signature obtained from MDA-MB-231 cells and found significant negative correlations (Supplementary Figure S5). Together these analyses suggest relatively robust reversal in drug response of MDA-MB-231 cells on induction of NME2 for three known anticancer molecules.

### Engineered reversal of drug resistance in cancer cells

**Figure 4.**
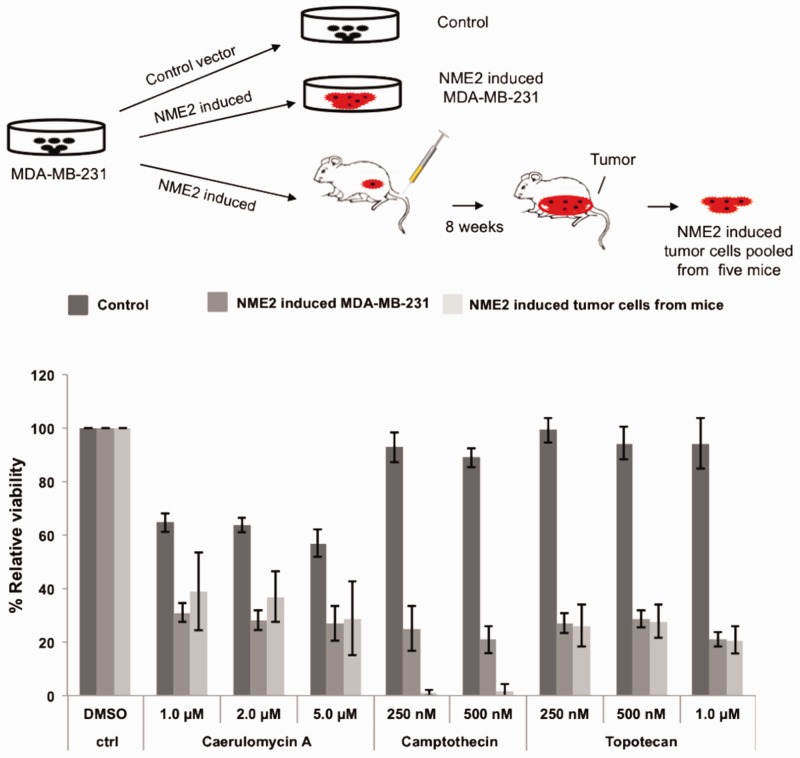
MSG-induction leads to reversal of drug resistance. Viability of cells in presence of anticancer molecules decreased after induction of NME2 in MDA-MB-231 cells, but not in un-induced control cells in both, under culture conditions and also when tumors were developed *in vivo* in mice (*n* = 5).

Next we tested the effect of NME2-induction on chemoresistance (Figure 4, upper panel). Stable MDA-MB-231 lines generated after induction of NME2 were treated with caerulomycin, camptothecin or topotecan, a derivative of camptothecin. For multiple camptothecin and topotecan concentrations we found that relative viability of cancer cells decreased by more than 60% in NME2-induced cells relative to cells with control vector, demonstrating increased sensitivity (Figure 4). Similarly treatment with caerulomycin resulted in 50% decrease in cell viability in NME2-induced MDA-MB-231 cells compared with vector control (Figure 4). To further test this effect *in vivo*, we developed tumors from NME2-induced cells in immune compromised mice. Cells extracted from the developed tumors after 8 weeks were again subjected to the chemosensitivity test. Consistent with the *in vitro* findings, cells extracted from tumors also showed increased chemosensitivity relative to control cells indicating that the NME2-induced effects are sustained within the tumor microenvironment (Figure 4).

Similarly, we induced NME2 expression in MDA-MB-468 breast cancer cells, which are also known to be drug resistant though not to the extent noted from MDA-MB-231 cells (31). Stable lines with induced expression of NME2 were treated with camptothecin or topotecan as done earlier. Once again we found that viability of cancer cells decreased by 20–25% in NME2-induced cells relative to control cells showing increased response toward the drug molecule (Supplementary Figure S6). The effects were modest in MDA-MB-468 cells compared with MDA-MB-231 cells possibly because the viability/resistance of the MDA-MB-468 cell type is known to be inherently less in presence of many chemotherapeutic drugs (32).

### Mesenchymal to epithelial transformation as a molecular process underlying reversal of resistance in cancer cells

**Figure 5.**
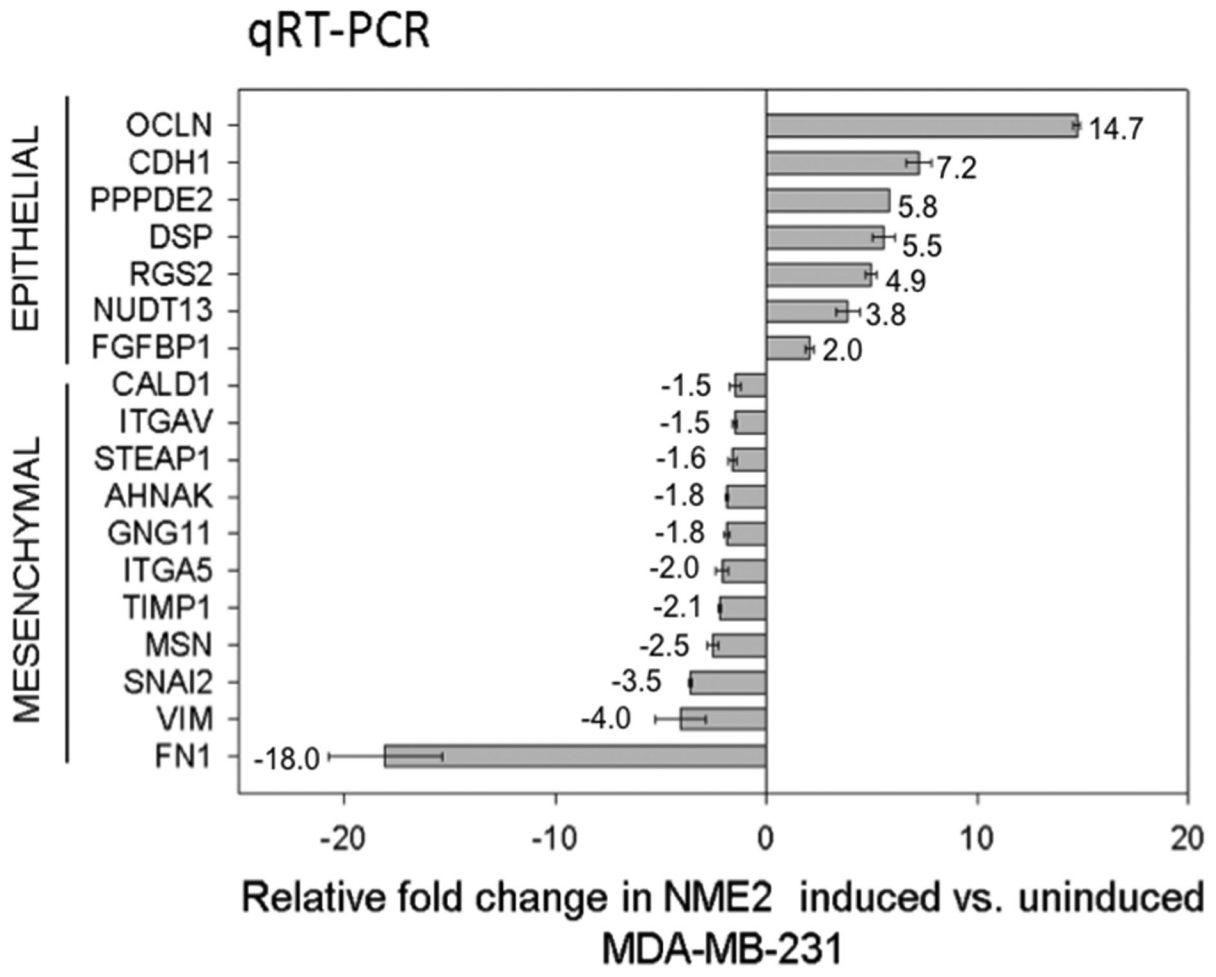
NME2 promotes MET. NME2-induced MDA-MB-231 cells showed up-regulation of epithelial and down-regulation of mesenchymal markers in quantitative real-time mRNA analysis.

Next, we checked whether the process of EMT was associated with NME2-mediated decrease in chemoresistance of cancer cells. As mentioned earlier, EMT has been linked to not only tumorigenesis but also increased drug resistance in cancer (1,12). We conjectured that increased sensitivity of cancer cells could be due to the opposing transformation, i.e., mesenchymal to epithelial transition (MET) on induction of NME2. Quantitative RT-PCR of NME2-induced MDA-MB-231 cells showed increase in several epithelial markers and decrease in mesenchymal markers suggesting induction of MET in MDA-MB-231 cells which are mesenchymal breast cancer cells with aggressive invasive potential (Figure 5). Induction of MET was also expected to lead to decreased invasiveness of the MDA-MB-231 cells. Both, decreased invasiveness (tested using modified Boyden chamber assays) and trans-endothelial migration, hallmarks of aggressive cancer cells was diminished on NME2-induction relative to un-induced cells (Supplementary Figure S7A and B). MDA-MB-468 cells also showed decreased invasiveness on NME2-induction relative to control cells (Supplementary Figure S7C).

To further test this approach we sought to test other MSGs. We found expression profiles following CLDN1 and RECK induction in CL1-5 and HT-1080, respectively (33). CLDN1 and RECK-specific ‘induction’ signatures were compared with the caerulomycin and camptothecin REM. In both cases we found negative correlation between the respective ‘induction’ signatures and camptothecin REM*_r_*_–_*_s_* (*P*-value 0.05; Supplementary Figure S8, upper panel). Following this we checked the genes involved in the induction signatures, several genes represented multiple pathways that modulate EMT signaling (Supplementary Figure S8, lower panel). Though further work is required, together these suggest that MET in cells with increased MSGs RECK and CLDN1 induce drug sensitivity.

In addition, we asked whether our approach was consistent with previously reported drug resistance studies in DrugBank (34) and Connectivity Map (7). For analysing DrugBank data on drugs and respective target/transporter we selected three drugs mitoxantrone, doxorubicin and camptothecin. Cells resistant or sensitive to these drugs were checked to first test whether EMT was activated in resistant cells using the well-established and robust Gene Set Enrichment Analysis (GSEA (35)). This confirmed that for all the three test cases genes involved in EMT were positively correlated with resistant cells (see Supplementary Figure S9). These findings are also in line with literature reports on expression of transporters (ABCG2 in case of mitoxantrone, doxorubicin and camptothecin) during EMT in drug resistant cancer cells (36,37). Next we analysed expression data from Connectivity Map for MCF7 cells and drugs doxorubicin, camptothecin and vinblastine. On performing the GSEA analysis for EMT genes we found genes involved in EMT were significantly enriched among differential expressed genes in resistant cells (Supplementary Figure S10).

We undertook the REM approach to test the hypothesis that factor(s) that modulate transcriptomes associated with drug response may also alter inherent cellular resistance when extraneously induced/repressed. This approach was tested over a group of cell lines implying its robustness, however with the added risk that too heterogeneous a cell group may result in non-specificity. Several features of the model suggest its ‘tunability’ across a range of gene/cell/drug combinations underscoring utility of the REM analysis in a variety of settings. For example, first, drug-specific REMs can be constructed for any chemosensitivity indicator of choice and stringency (e.g., by increasing/decreasing the GI50 cut-off). Once developed, a drug-REM can be used to screen against ‘induction’ or ‘repression signatures’ of any gene of interest. In addition, and perhaps more interestingly, ‘induction signatures’ developed for small molecule ligands can be used to predict molecules that augment chemosensitivity when administered in combination with a primary chemotherapeutic agent.

In this study we focused on identifying candidate MSGs, if any, which would impact drug resistance in cancer cells. The primary reason for this approach was the understanding that even one MSG could impact resistance/sensitivity of cancer cells. Our observation on considering all the 36 MSGs as a group—where significant correlation with resistance cells were found only in a subset of cases (59% of the drug–cell combinations studied by us) appears to be in line with the understanding. Furthermore, it also suggests that function of MSGs may be context specific (see below).

In this case study we focused on reversal of resistance. Based on the analysis showing REM*_r_*_–_*_s_* negatively correlates with the ‘induction signature’ of NME2 we induced a selected MSG. It must be noted that all MSGs may not produce similar results. In addition, MSGs depending on the cell type or physiological context may function distinctly. For example, NME2 is up-regulated in resistant cells in many cell–drug combinations (Supplementary Figure S1). The correlation, positive or negative, between REM versus ‘induction signature’ would determine how repression or induction of the MSG would influence chemosensitivity. For many MSG/cell/drug combinations repression co-segregated with sensitivity (Figure 2A), in such cases it is likely that repression, instead of induction, would lead to induced chemosensitivity for the drug molecule in question. The seemingly opposite activities of MSG with respect to chemosensitivity may be due to the contextual dependence on a combination of factors, including cellular physiology, mode of drug action and biological pathways that determine drug sensitivity. Keeping these in mind, in order to select a candidate for this case study we adopted an approach that involved analysis of patient samples (Supplementary Figure S3).

It is likely that other MSGs also have similar functions. To prioritize selection of any MSG for experimental testing, expression profiles following induction or repression of the MSG can be compared with drug-specific REM*_r_*_–_*_s_*. The resultant correlation value is expected to be a reasonable indicator of false discovery. Further support for the selection can be gained from analysis of genes involved in the induction signature; for example, we found several genes represented multiple pathways that modulate EMT signaling. Together these would increase the potential of finding other MSGs that induce chemosensitivity in a cell/drug-specific manner.

It is possible that all MSGs do not influence metastasis potential in similar ways (38). Particularly since metastasis involves a number of stages/processes, which may in principle be antagonistic—for example, EMT at the inception of the metastatic process may need to be complemented with MET at the distant site for effective colonization of cells following extravasation into the parenchyma. Thus, regardless of the underlying biological pathways, REM being derived from gene expression profiles that portray the end-point of a combination of physiological events, gives a robust prediction method for identifying suitable factors that can be tested for reversal of chemoresistance.

## SUPPLEMENTARY DATA

Supplementary Data are available at NAR Online, including [39–49].

Supplementary Data
