# Supplementary material for: Engineered reversal of drug resistance in cancer cells—metastases suppressor factors as change agents
Source: Nucleic Acids Res. 2013 Oct 23;42(2):764–73. doi: 10.1093/nar/gkt946 (PMC3902936; doi:10.1093/nar/gkt946)
Supplement: Supplementary Data [file supp_42_2_764__index.html]

Engineered reversal of drug resistance in cancer cells—metastases suppressor factors as change agents — Engineered reversal of drug resistance in cancer cells—metastases suppressor factors as change agents — Supplementary Data 

# Engineered reversal of drug resistance in cancer cells—metastases suppressor factors as change agents

## Supplementary Data

files

**Files in this Data Supplement:**

- Supplementary Data - pdf file
