# Supplementary material for: Engineered reversal of drug resistance in cancer cells—metastases suppressor factors as change agents
Source: Nucleic Acids Res. 2013 Oct 23;42(2):764–73. doi: 10.1093/nar/gkt946 (PMC3902936; doi:10.1093/nar/gkt946)
Supplement: Supplementary Data [file supp_gkt946_nar-02046-n-2013-File002.pdf]

Supplementary Figure 1

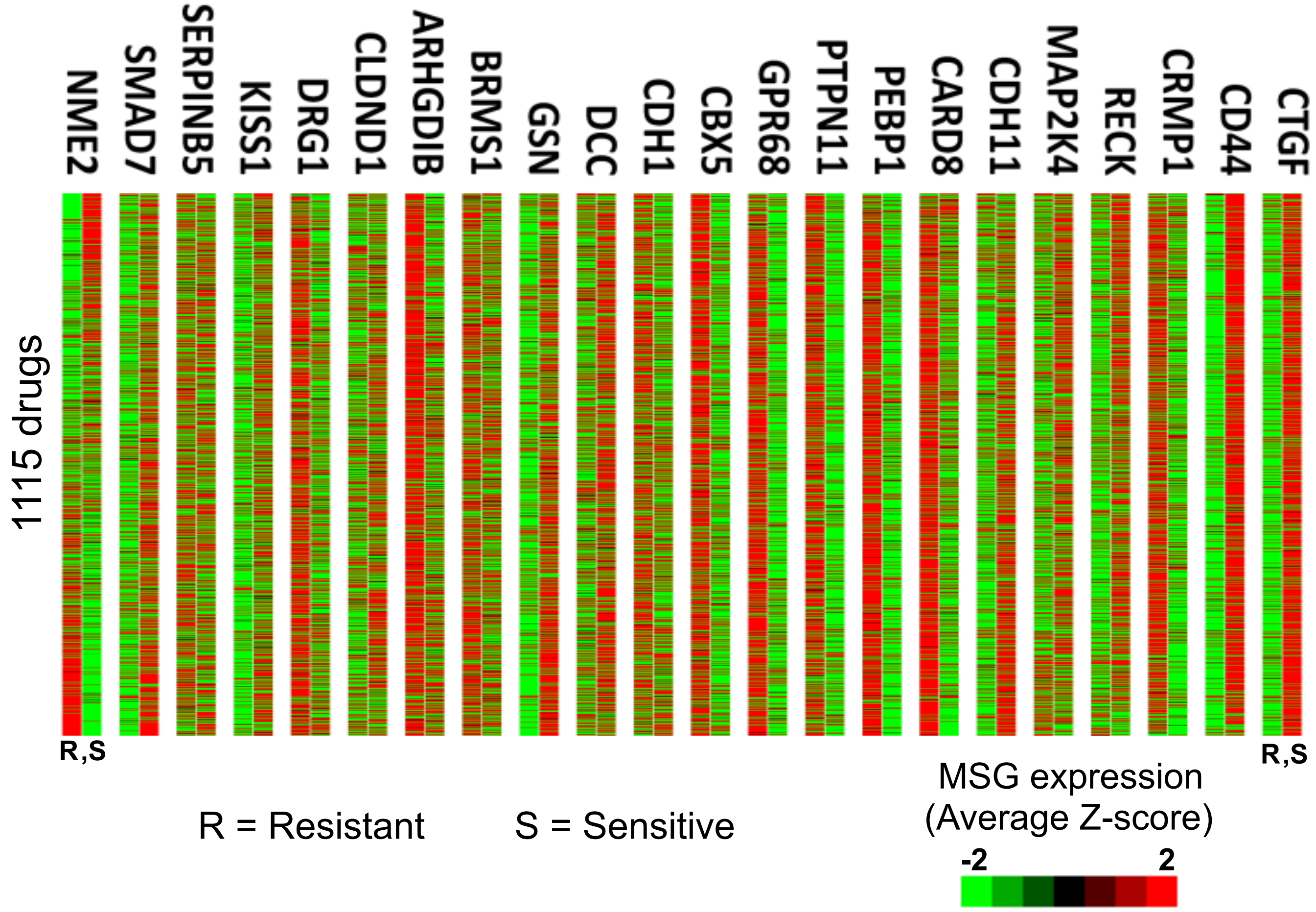

Supplementary Figure 1. For each of the 1115 drugs and corresponding cell-groups expression of the MSG in resistant versus sensitive cells was compared. Several MSG's were down-regulated in resistant cell lines and conversely up-regulated in sensitive cells for many drugs, and vice versa

Supplementary Figure 2

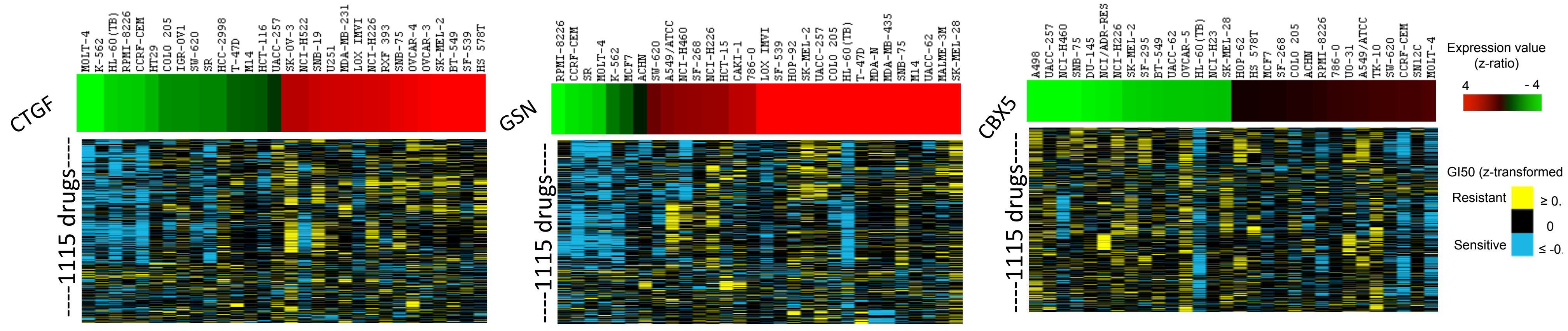

Supplementary Figure 2. Cell lines with high or low expression of the MSG show distinct response to many drug molecules.

Supplementary Figure 3

A)

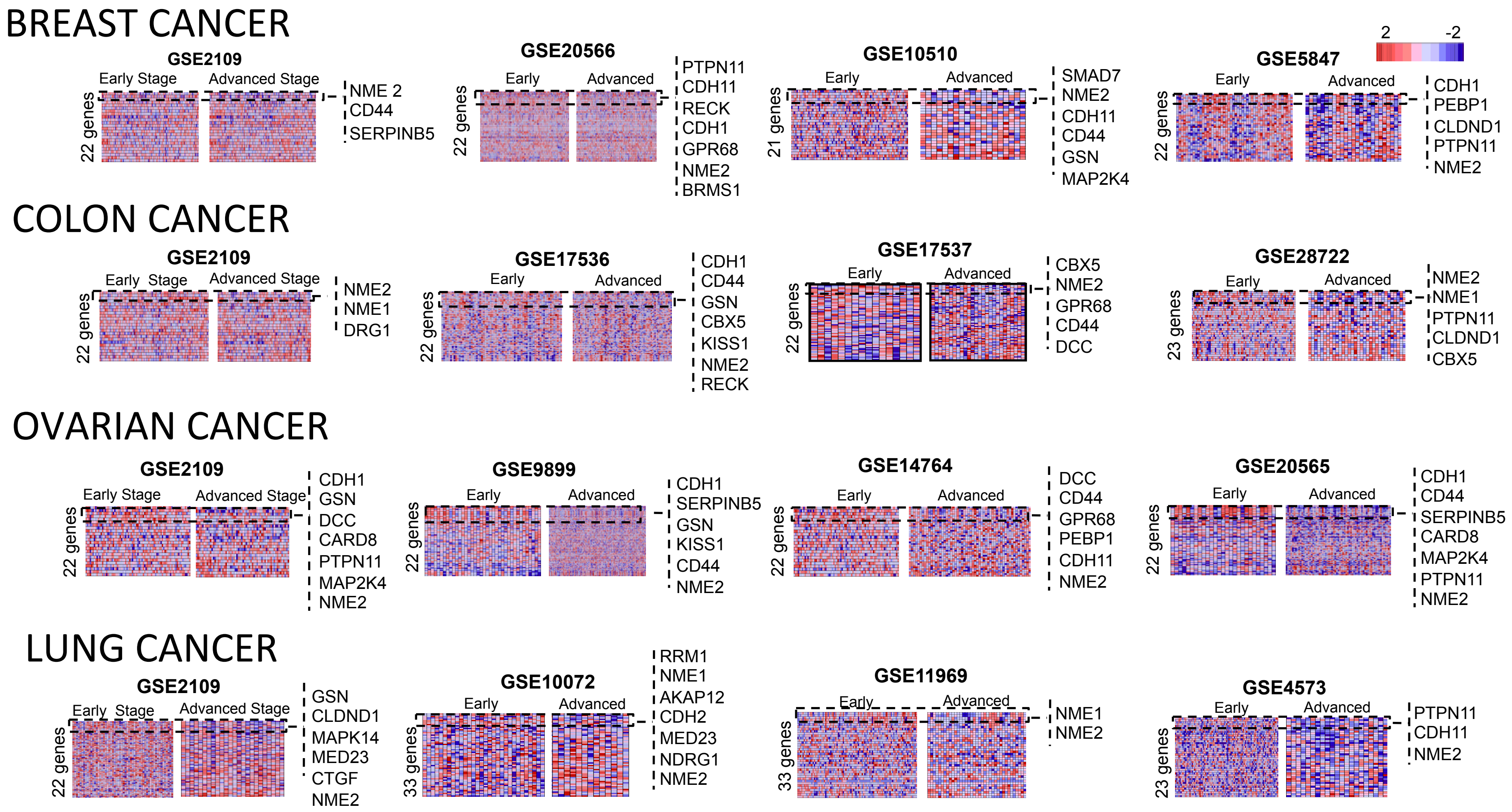

B)

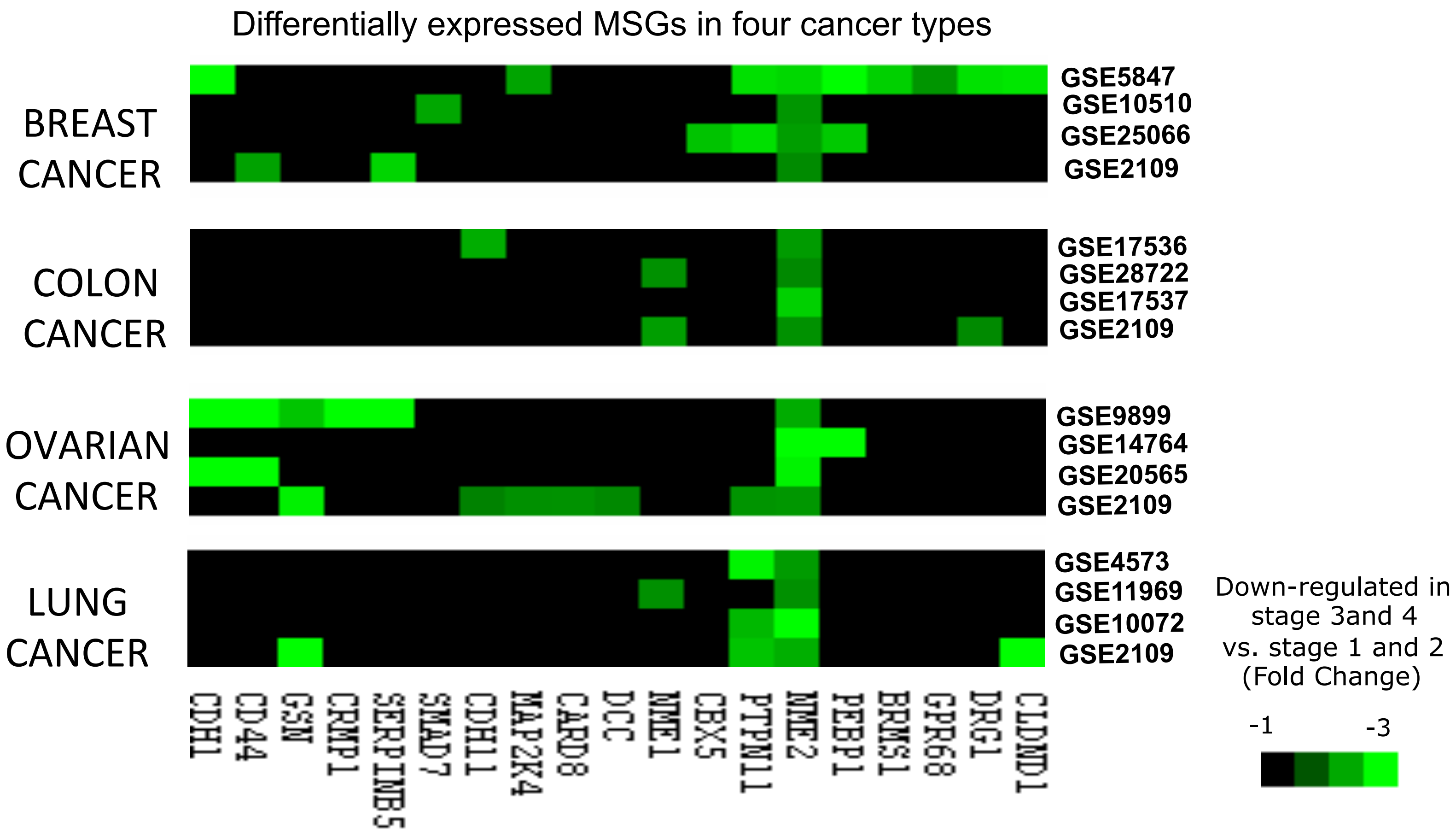

Supplementary Figure 3.(A) Analysis from independent clinical cohorts of breast, colon, ovarian and lung cancer - right panels show MSGs with reduced expression in advanced (stage 3 and 4) relative to early stages (stage 1 and 2); expression index: red, up-regulation; blue, down-regulation. **(B)** Heat map of 19 MSGs with reduced expression in advanced stages in at least one data set; scale: fold change of expression

Supplementary Figure 4

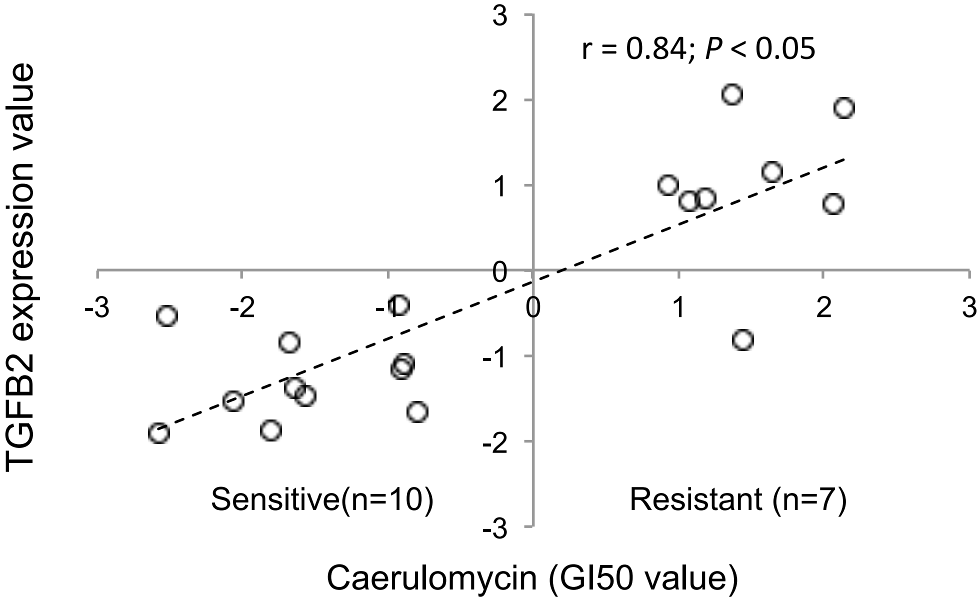

Supplementary Figure 4. Expression of the TGFB2 is correlated with increase in GI50 of towards caerulomycin

Supplementary Figure 5

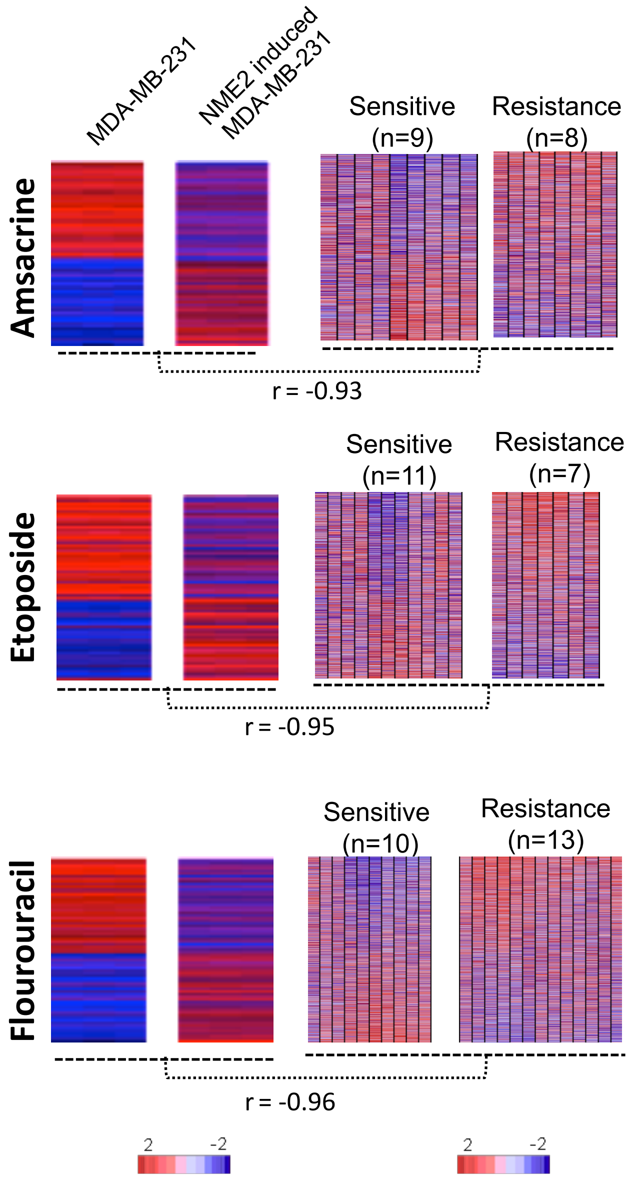

Supplementary Figure 5.Reversal of the resistance signature following MSG induction. Comparative analysis of the 'MSG-induction' signature versus drug-specific response-engineering-module (REM).NME2 expression in cell lines grouped as resistant or sensitive is shown in left panel. Expression profiling of MDAMB-231 cells before and after NME2 induction and the resultant change in transcriptome of MDAMB-231 cells is compared with the sensitive/resistant gene signature derived for either Amsacrine, Etoposide or Flourouracil**.** Correlation of the resistant-minus-sensitive gene signature derived from multiple cell lines – response engineering module (REM_{r-s}) – with the 'NME-induction' signature in MDAMB231 cells is mentioned at bottom of each heat map.

Supplementary Figure 6

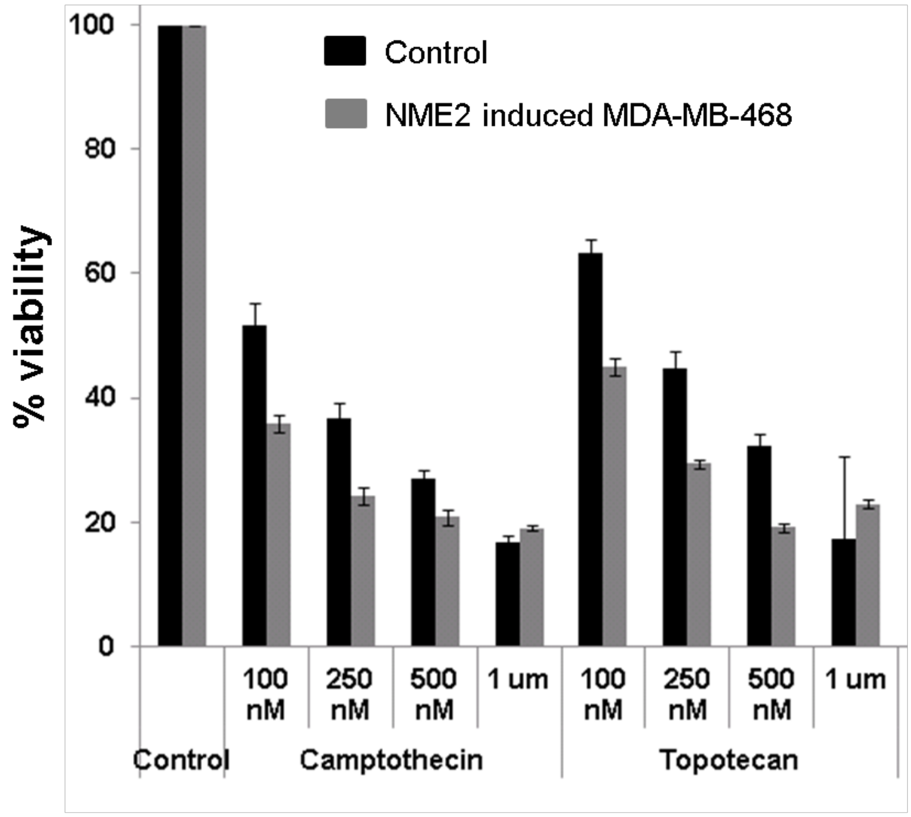

Supplementary Figure 6. MSG-induction leads to reversal of drug resistance in MDAMB-468 cells. Viability of cells in presence of anticancer molecules decreased in NME2-induced MDAMB-468 cells, but not in un-induced control cells.

Supplementary Figure 7

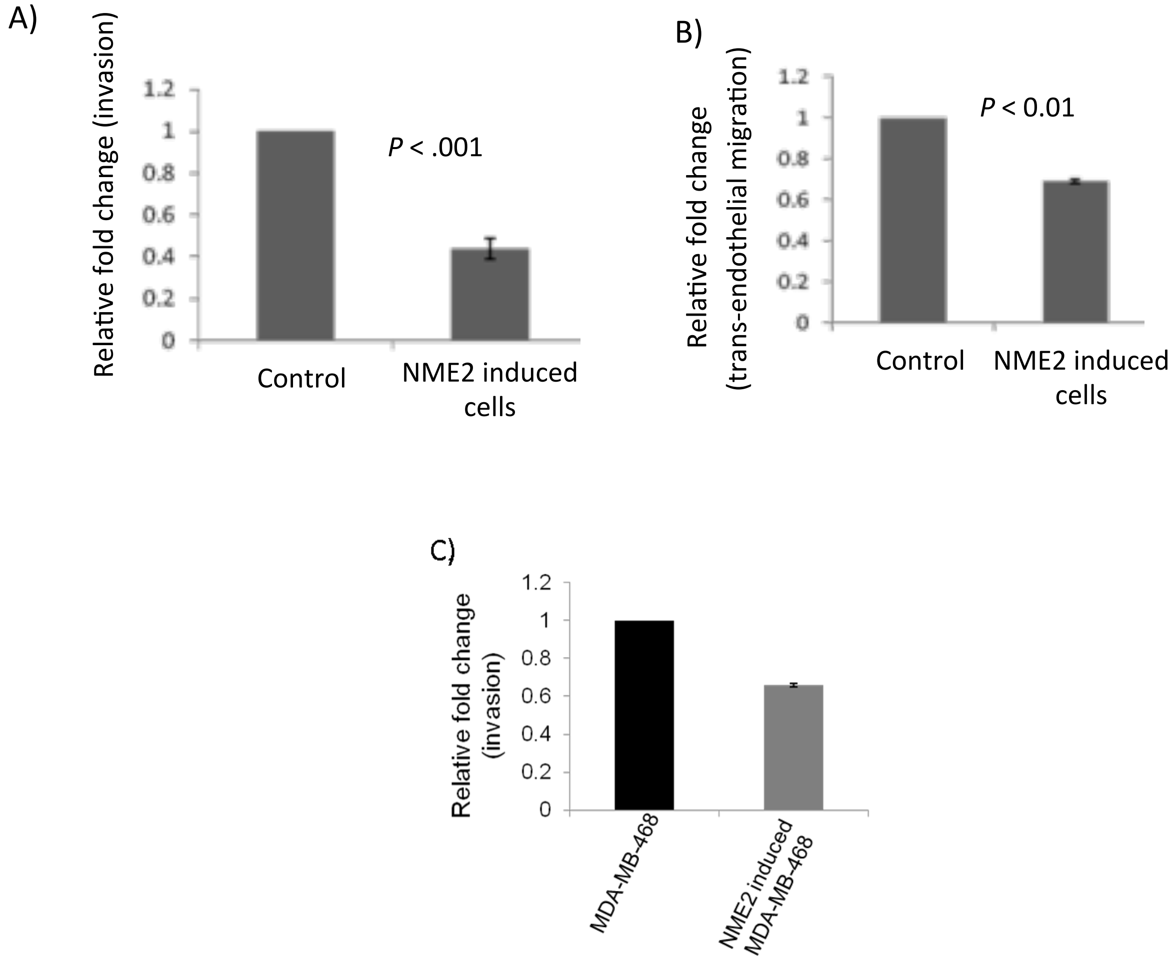

Supplementary Figure 7. NME2 induced MDA-MB-231 cells show decreased invasiveness (A) and trans-endothelial migration (B). Invasion in NME2-induced MDAMB-468 relative to control cells (C)

Supplementary Figure 8

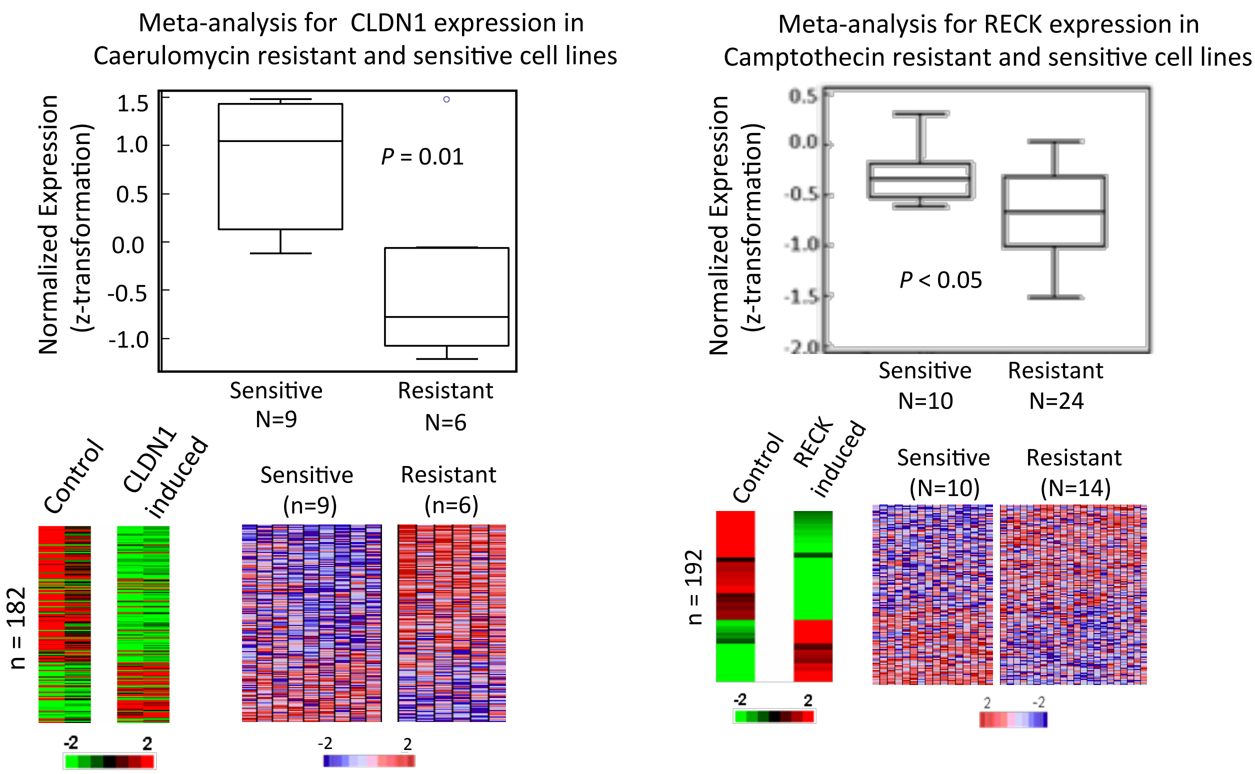

Pathways	Corrected P-Value
Cell adhesion	0.01
TGF-beta signaling	0.03
PPAR signaling	0.03
JAK-STAT cascade	0.04
Notch signaling	0.05

Pathways	Corrected P-Value
TGF-beta signaling	0.005
VEGF signaling	0.01
JAK-STAT cascade	0.01
Cell-matrix adhesion	0.02
Wnt receptor signaling	0.04

Supplementary Figure 8. Profiles ofCLDN1 and RECK induction in CL1-5 and HT-1080 cells respectively. CLDN1 and RECK and specific ‘induction’ signatures, respectively were compared with the caerulomycin and camptothecin $REM_{r\text{-}s}$. Pathway analysis for genes of CLDN1 and RECK gene signature.

Supplementary Figure 9

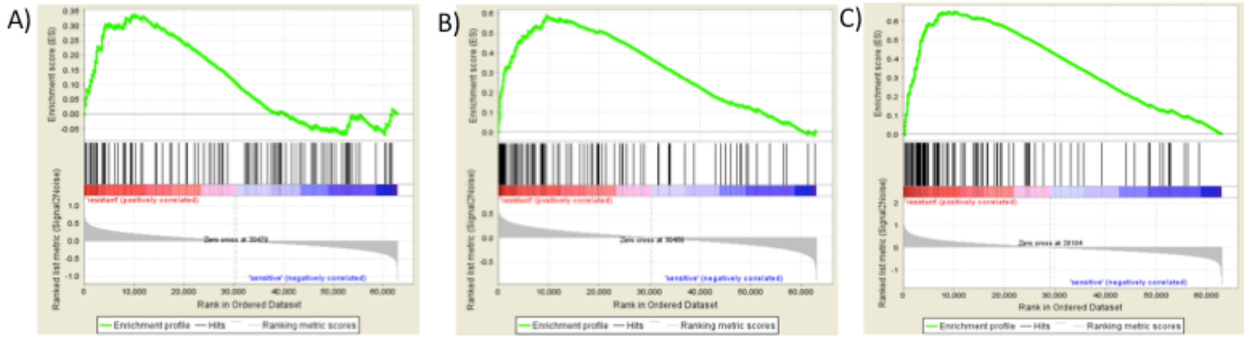

Supplementary Figure 9: Gene set enrichment analysis (GSEA) was performed with genes up regulated in EMT for Mitoxantrone (A), Camptothecin (B) and Doxorubicin (C). "Signal-to-Noise" ratio (SNR) statistic was used to rank the genes according to their correlation with either the resistant phenotype (red) or sensitive phenotype (blue). The graph on the bottom of each panel represents the ranked, ordered, non-redundant list of genes. Genes on the far left (red) correlated the most with resistant cell lines, and genes on the far right (blue) correlated the most with sensitive cell lines. On each panel, the vertical black lines indicate the position of each of the genes of the studied gene set in the ordered, non-redundant data set. The green curve corresponds to the ES (enrichment score) curve, which is the running sum of the weighted enrichment score obtained from GSEA.

Supplementary Figure 10

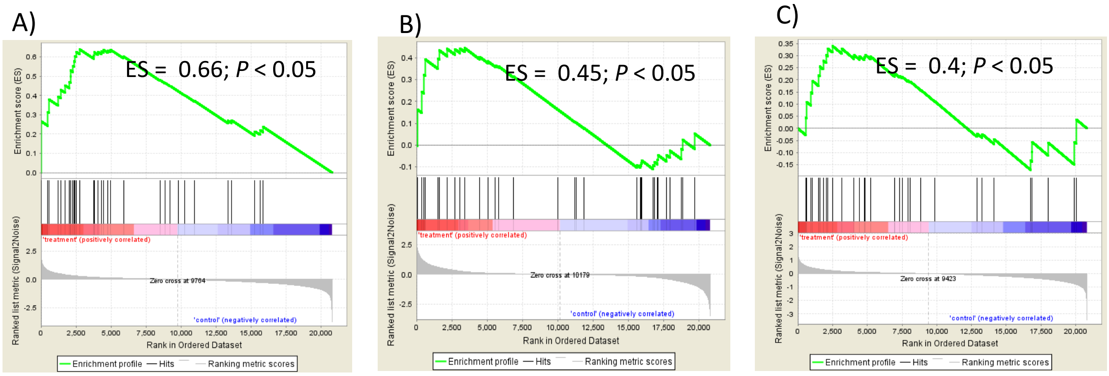

Supplementary Figure 10: Gene set enrichment analysis (GSEA) was performed with genes up regulated in EMT for Camptothecin (A), Vinblastine (B) and Doxorubicin (C). Expression profile was downloaded from Connectivity Map where MCF7 cells transcriptome study was done after drug treatments.

Supplementary Methods

Meta-analysis of clinical transcriptome profile

To identify differentially expressed MSGs in lung, ovary, colon and breast cancer, different expression datasets were used. For expression datasets of these cancers, expression profile generated from clinical samples at different stages were downloaded from Gene Expression Omnibus (GEO). From the GEO database the expression studies that were downloaded for the clinical study of MSGs were GSE2109 (http://www.intgen.org/expo/), GSE25055 (39), GSE10510 (40), GSE5847 (41), GSE17536 (42), GSE17537 (42), GSE28722 (43), GSE9899 (44), GSE14764 (45), GSE20565 (46), GSE10072 (47), GSE11969 (48), GSE4573 (49).

For normalization of raw intensity provided in these expression studies, Z-score transformation method was used where raw intensity data for each experiment is log10 transformed. Z scores were calculated by subtracting the overall average gene intensity (within a single experiment) from the raw intensity data for each gene, and dividing that result by the SD of all of the measured intensities, according to the formula:

$$\text{Z score} = (\text{intensity}_{G} \text{ -mean intensity}_{G1...Gn})/\text{SD}_{G1...Gn}$$

Where G is any gene on the microarray and G1. . . Gn represents the aggregate measure of all of the genes.

Each dataset was first grouped to be in either early or the advanced stage and difference in MSG expression was measured between the two stages. To identify significant differentially expressed genes two-tailed student’s t-test was used and *P*-value cutoff at 0.05 was applied. MSGs with an down-regulation in advance stage were identified.

39. Hatzis, C., Pusztai, L., Valero, V., Booser, D.J., Esserman, L., Lluch, A., Vidaurre, T., Holmes, F., Souchon, E., Wang, H. *et al.* (2011) A genomic predictor of response and survival following taxane-anthracycline chemotherapy for invasive breast cancer. *JAMA*, **305**, 1873-1881.

40. Calabro, A., Beissbarth, T., Kuner, R., Stojanov, M., Benner, A., Asslaber, M., Ploner, F., Zatloukal, K., Samonigg, H., Poustka, A. *et al.* (2009) Effects of infiltrating lymphocytes and estrogen receptor on gene expression and prognosis in breast cancer. *Breast Cancer Res.Treat.*, **116**, 69-77.

41. Boersma, B.J., Reimers, M., Yi, M., Ludwig, J.A., Luke, B.T., Stephens, R.M., Yfantis, H.G., Lee, D.H., Weinstein, J.N. and Ambs, S. (2008) A stromal gene signature associated with inflammatory breast cancer. *Int.J.Cancer*, **122**, 1324-1332.

42. Smith, J.J., Deane, N.G., Wu, F., Merchant, N.B., Zhang, B., Jiang, A., Lu, P., Johnson, J.C., Schmidt, C., Bailey, C.E. *et al.* (2010) Experimentally derived metastasis gene expression profile predicts recurrence and death in patients with colon cancer. *Gastroenterology*, **138**, 958-968.

43. Loboda, A., Nebozhyn, M.V., Watters, J.W., Buser, C.A., Shaw, P.M., Huang, P.S., Van't, V.L., Tollenaar, R.A., Jackson, D.B., Agrawal, D. *et al.* (2011) EMT is the dominant program in human colon cancer. *BMC.Med.Genomics*, **4**, 9.

44. Tothill, R.W., Tinker, A.V., George, J., Brown, R., Fox, S.B., Lade, S., Johnson, D.S., Trivett, M.K., Etemadmoghadam, D., Locandro, B. *et al.* (2008) Novel molecular subtypes of serous and endometrioid ovarian cancer linked to clinical outcome. *Clin.Cancer Res.*, **14**, 5198-5208.

45. Denkert, C., Budczies, J., rb-Esfahani, S., Gyorffy, B., Sehouli, J., Konsgen, D., Zeillinger, R., Weichert, W., Noske, A., Buckendahl, A.C. *et al.* (2009) A prognostic gene expression index in ovarian cancer - validation across different independent data sets. *J.Pathol.*, **218**, 273-280.

46. Meyniel, J.P., Cottu, P.H., Decraene, C., Stern, M.H., Couturier, J., Lebigot, I., Nicolas, A., Weber, N., Fourchotte, V., Alran, S. *et al.* (2010) A genomic and transcriptomic approach for a differential diagnosis between primary and secondary ovarian carcinomas in patients with a previous history of breast cancer. *BMC.Cancer*, **10**, 222.

47. Landi, M.T., Dracheva, T., Rotunno, M., Figueroa, J.D., Liu, H., Dasgupta, A., Mann, F.E., Fukuoka, J., Hames, M., Bergen, A.W. *et al.* (2008) Gene expression signature of cigarette smoking and its role in lung adenocarcinoma development and survival. *PLoS.One.*, **3**, e1651.

48. Takeuchi, T., Tomida, S., Yatabe, Y., Kosaka, T., Osada, H., Yanagisawa, K., Mitsudomi, T. and Takahashi, T. (2006) Expression profile-defined classification of lung adenocarcinoma shows close relationship with underlying major genetic changes and clinicopathologic behaviors. *J.Clin.Oncol.*, **24**, 1679-1688.

49. Raponi, M., Zhang, Y., Yu, J., Chen, G., Lee, G., Taylor, J.M., Macdonald, J., Thomas, D., Moskaluk, C., Wang, Y. *et al.* (2006) Gene expression signatures for predicting prognosis of squamous cell and adenocarcinomas of the lung. *Cancer Res.*, **66**, 7466-7472.
